# Impact of *Spirulina* Supplementation on Obesity, Hypertension, Hyperglycemia, and Hyperlipidemia: A Systematic Review

**DOI:** 10.1155/sci5/6637793

**Published:** 2025-03-27

**Authors:** Muhamad Firdaus, Ahmad Faris Priambodo

**Affiliations:** Department of Fisheries and Marine Resources Management, Faculty of Fisheries and Marine Science, Universitas Brawijaya, Malang 65145, Indonesia

**Keywords:** dietary supplements, hyperglycemia, hyperlipidemia, hypertension, obesity, spirulina, systematic review

## Abstract

**Aims:** This systematic review evaluates how *Spirulina* supplementation impacts human metabolic syndrome. This review encompasses a broad spectrum of *Spirulina* supplementation studies, including variations in dosage, trial duration, participants, and the subsequent effects on human metabolic syndrome.

**Methods:** The method involves searching for articles from the Scopus and PubMed databases (up to 2023) to identify relevant studies on *Spirulina* supplementation. The journal database related to the study was examined using the systematic review approach.

**Results:** Out of 527 articles related to *Spirulina* supplementation, 13 articles were determined to be suitable for human clinical studies. The treatment is performed at 20 mg to 6 g within 17–360 days. *Spirulina* contains macro- and micronutrients, vitamins, and minerals that are beneficial for health, as well as several bioactives that play a role in improving metabolic syndrome. This seaweed pigment can reduce obesity, body mass index (BMI), hypertension, hyperglycemia, and dyslipidemia. Its tripeptides lower blood pressure while gamma linoleic acid and glycolipids improve lipid profile. The findings show that *Spirulina* supplementation improves human metabolic syndrome. It included obesity, hypertension, hyperglycemia, and hyperlipidemia.

**Conclusion:** *Spirulina* supplementation in humans has a positive effect on metabolic syndrome. It is due to phycocyanin, L-isoleucyl-L-glutaminyl-L-proline (Ile-Gln-Pro), gamma-linolenic acid, and the glycolipid H-b2. However, the form of use, dosage, and treatment should be further researched to determine the right amount for each metabolic syndrome problem.

## 1. Introduction

Metabolic syndrome poses a significant public health concern because of its increasing incidence globally and its association with serious life-threatening illnesses [1, 2]. Many metabolic risk factors in the same individual characterize the metabolic syndrome [2, 3]. The initial unified agreement about the definition of metabolic syndrome was drawn up during a meeting hosted by the International Diabetes Federation in 2005. As stated in this, the main contributing factor is obesity, determined by waist circumference and BMI [3]. The prevalence of metabolic syndrome depends on age, gender, race, and ethnicity worldwide, ranging from 10% to 84%. It is estimated that 20%–25% of the world's adult population, including Indonesian people, have metabolic syndrome [2].

The contributing factors that cause metabolic syndrome are internal and external to the human body. Internal means genes or hereditary, whereas external means lifestyle [2, 3]. Describing lifestyle factors is eating unhealthy food. Patients with metabolic syndrome must avoid food and beverages with high sugar (sucrose and fructose), fat, and carbohydrate contents [4]. Metabolic syndrome has multiple underlying causes [2, 3]. These risk factors are called metabolic syndrome, including abdominal obesity, increased triglyceride levels, decreased high-density lipoprotein (HDL) cholesterol concentrations, elevated blood pressure, and hyperglycemia [5]. Metabolic syndrome increases the risk of cardiovascular disease, Type 2 diabetes, cancer (liver, pancreas, breast, and bladder), kidney dysfunction, pancreatic dysfunction, coronary or peripheral atherosclerosis, and heart failure [2, 3]. Moreover, metabolic syndrome is associated with several other systemic issues that impact many organs and systems, including fatty liver disease, respiratory disorders, osteoarthritis, and cancer [3].

Metabolic syndrome and obesity are closely linked, with each condition often exacerbating the other. Obesity, characterized by excessive accumulation of body fat, is a significant risk factor for developing metabolic syndrome, a group of disorders that include high blood pressure, high blood sugar, excess body fat around the waist, and abnormal cholesterol levels. The excess adipose tissue in obese individuals contributes to insulin resistance, inflammation, and dyslipidemia, which are critical components of metabolic syndrome. Conversely, the presence of metabolic syndrome can further promote weight gain and obesity by disrupting normal metabolic processes and increasing appetite. This bidirectional relationship highlights the importance of treating both conditions simultaneously to reduce the risk of associated health complications such as cardiovascular disease and Type 2 diabetes [4, 5].

The connection between metabolic syndrome and hypertension is significant because hypertension is a common component and consequence of metabolic syndrome. Metabolic syndrome encompasses a group of disorders, including insulin resistance, central obesity, dyslipidemia, and elevated blood glucose levels, all of which contribute to the development and exacerbation of hypertension. Insulin resistance and obesity, in particular, lead to several physiological changes, such as increased sympathetic nervous system activity, sodium retention, and changes in blood vessel function that increase blood pressure. Additionally, the inflammatory condition associated with metabolic syndrome can damage blood vessels, promoting artery stiffness and further increasing blood pressure. Effective treatment of metabolic syndrome through lifestyle changes and medical interventions is crucial for controlling high blood pressure and reducing the risk of cardiovascular events and other related health problems [2, 4].

Hyperglycemia is a critical component of metabolic syndrome. Metabolic syndrome is characterized by some disorders, including insulin resistance, central obesity, dyslipidemia, hypertension, and elevated fasting blood glucose levels. Insulin resistance, a hallmark of metabolic syndrome, impairs the body's ability to use glucose effectively, leading to elevated blood sugar levels or hyperglycemia. Chronic hyperglycemia, in turn, increases insulin resistance and contributes to further metabolic disorders. The interaction of these diseases creates a vicious circle that can lead to the development of Type 2 diabetes and increase the risk of cardiovascular disease. Managing hyperglycemia through lifestyle interventions such as diet, exercise, and medical treatments is critical to breaking this cycle and mitigating the health impairments associated with metabolic syndrome [1, 3, 5].

The relationship between metabolic syndrome and hyperlipidemia is complicated and mutually reinforcing. Hyperlipidemia, characterized by increased levels of lipids in the blood, including cholesterol and triglycerides, is a common feature of metabolic syndrome. Metabolic syndrome includes a range of conditions, such as insulin resistance, central obesity, hypertension, and hyperglycemia, all of which contribute to lipid abnormalities. In particular, insulin resistance and obesity lead to increased production of very low-density lipoprotein (VLDL) and decreased clearance of triglycerides from the bloodstream, leading to hyperlipidemia. In addition, metabolic syndrome is often accompanied by a proinflammatory condition that can further disrupt lipid metabolism. This dyslipidemia increases the risk of atherosclerosis and cardiovascular disease, which is why control of lipid levels is crucial in treating metabolic syndrome. Addressing hyperlipidemia through lifestyle changes, dietary modifications, and pharmacological interventions is critical for reducing cardiovascular risks associated with metabolic syndrome [3, 5].

Macronutrients and micronutrients are essential in protecting the body against metabolic syndrome. Carbohydrates, lipids, and proteins are the body's main energy sources. Elevated blood sugar levels after consuming simple carbohydrates can raise the risk of metabolic syndrome. People with metabolic syndrome are recommended to choose complex carbohydrates and reduce their intake of simple carbohydrates, ingest enough protein, and choose to consume mono- and polyunsaturated fatty acids [6]. Vitamins and minerals are nutrients that the body needs in trace levels. Micronutrients are vital in protecting the body from metabolic syndrome. Vitamin and mineral deficiencies in vitamins B complex, C, and D, and Mg and K can elevate the risk of metabolic syndrome [7].

*Spirulina* is frequently utilized as a dietary supplement because it has the characteristics of high nutrients and bioactive components and is safe for human consumption [8–10]. Approximately 55%–69% proteins, 6%–7% lipids (rich in polyunsaturated fatty acids, or PUFAs), 15%–24% carbohydrates, and minerals and antioxidants are all present in high concentrations in *Spirulina* [11]. Several micronutrients are rich in *Spirulina*, including beta-carotene, B vitamins, and minerals [12]. *Spirulina* also has phycocyanin as a major pigment with significant advantages for humans [10, 13].

*Spirulina* is a blue-green microalgae from phylum *Cyanobacteria* that flourishes in aquatic environments, and species of *Spirulina* have been isolated from tropical waters to the North Sea [8, 10]. The most well-known species of *Spirulina* in other studies are *S. platensis*, *S. maxima*, and *S. fusiformis* [8, 13]. *Spirulina*, a nutrient-dense cyanobacterium, shows significant potential as a complementary therapy for chronic conditions such as obesity, diabetes, and dyslipidemia. It aids in weight reduction by decreasing body weight, fat percentage, and waist circumference while controlling appetite through phenylalanine-induced release of cholecystokinin and improved leptin sensitivity. *Spirulina* enhances glycemic control and insulin sensitivity by increasing insulin production, improving hemoglobin levels, and reducing inflammatory mediators. Additionally, it improves lipid profiles by lowering total cholesterol and triglycerides through its action on HMG-CoA reductase and lipid metabolism. Another mechanism for improving the lipid profile of *S. platensis* is the inhibition of HMG-CoA reductase activity and activation of the lecithin cholesterol acyl transferase (LCAT) enzyme [14]. Its anti-inflammatory and antioxidant properties, attributed to compounds like C-phycocyanin and β-carotene, reduce oxidative stress and inflammation. *Spirulina* high-protein content further aids in weight management and cardiovascular health by reducing oxidative stress and managing endothelial dysfunction [9, 11, 13, 15–17].

Several clinical studies have examined the effects of *Spirulina* on food intake and energy expenditure. A double-blind, placebo-controlled study in 52 obese adults found that 12 weeks of spirulina supplementation significantly reduced food intake and body weight compared with placebo [18]. This decrease in food intake is associated with increased release of the hormone GLP-1, which induces satiety. Another study in 40 athletes showed that spirulina supplementation for four weeks increased energy expended during exercise compared to placebo [19]. This increase in energy expenditure correlates with increased fat oxidation, indicating a potential effect of spirulina in improving metabolism. In a clinical trial on 78 Type 2 diabetes patients, spirulina supplementation for 12 weeks significantly reduced food intake and body weight [20]. This effect is attributed to the inhibition of appetite by spirulina.

*Spirulina* has been studied in combination with conventional drugs related to metabolic syndrome, such as antidiabetics [21]. In a study on streptozotocin-induced diabetic rats, spirulina showed comparable hypoglycemic effects to glibenclamide, a sulfonylurea antidiabetic drug [22]. When combined, *Spirulina* and glibenclamide produced a more significant reduction in blood glucose than either treatment alone. A clinical trial in patients with Type 2 diabetes found that spirulina supplementation improved glycemic control and insulin sensitivity when added to metformin [23]. With many uses of *Spirulina* as a dietary supplement, this systematic review aims to get an overview of the effect of supplementation on human-specific metabolic syndrome.

## 2. Method

The method used for this review is a systematic review using references from Khan et al. [24]. The systematic review strategy begins with a clearly stated problem, locates relevant research, rates the quality of those studies, and uses a clear methodology to compile the evidence. Systematic reviews are different from other reviews and commentaries because of their clear and rigorous process [25].

The method used in reviewing articles is a systematic review using PubMed and Scopus as article search engines and Mendeley as the article citation software. The steps and method of gathering data utilized in this review are information extraction from a database of the articles with the following details: (1) open the article search engine and then try to create keywords according to the desired topic limits, (2) set the desired year period, (3) download the database obtained, (4) input into Mendeley software, (5) screen each article and determine eligibility criteria, (6) carry out inclusion and exclusion according to the desired topic, (7) for articles included in the inclusion criteria, data are tabulated using the following information criteria:

a. Author's name
b. Year of publication
c. Location of research
d. Design of research
e. Duration of trial
f. Inclusion criteria
g. Species of sample
h. Dose of sample
i. Participant
j. Age of participant
k. Results of research

(8) include more in-depth research results from each article in the results section, and (9) discuss in more depth the discussion of each article and additions from other more concrete articles following the discussion.

### 2.1. Search Strategy and Study Selection

The search engines employed in this review are PubMed and Scopus with up –2023 periods. The surveying article's topic is *Spirulina*'s effect on human metabolic syndrome. The search code that was used is (*Spirulina* OR Arthrospira) AND (obes⁣^∗^ OR “body mass index” OR BMI OR “waist circumference” OR “hip circumference” OR “waist to hip ratio” OR WHR OR anthropomet⁣^∗^ OR “blood pressure” OR hypertension OR “serum triglyceride” OR “serum low density lipoprotein cholesterol” OR “serum high density lipoprotein cholesterol” OR LDL-C OR HDL-C OR “serum total cholesterol” OR “insulin resistance” OR “fasting blood glucose” OR FBS OR “blood glucose” OR diabet⁣^∗^ OR “metabolic syndrome”). The clinical human trials are included in this evaluation. After obtaining the pertinent studies from the article, it will be citations by using the software Mendeley.

### 2.2. Eligibility Criteria

The eligibility requirements are all studies with clinical trials that employed *Spirulina* to examine the impact on the metabolic human system. The qualifying criteria for inclusion and exclusion of the studies refer to participants, intervention, comparator, and outcomes (PICO), and it is displayed in Table 1.

## 3. Result

### 3.1. Search Result

**Figure 1:**
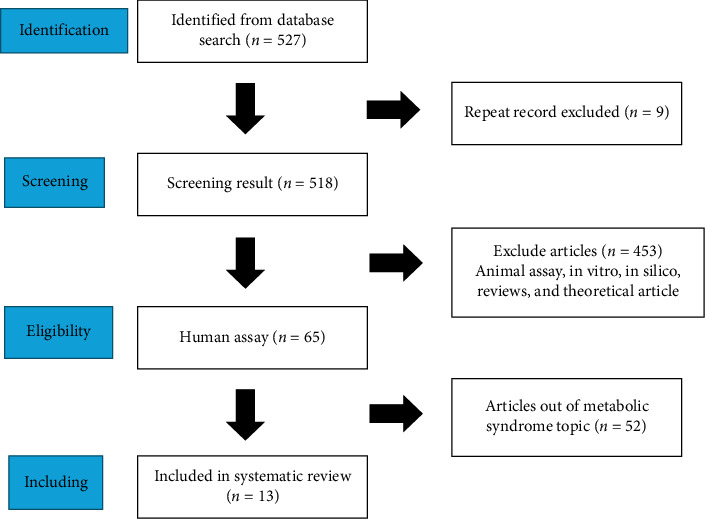
Systematic review study scheme.

After searching the article from the database that has been determined, the next step is to perform screening by analyzing the inclusion criteria of each article against the specified review topics in Figure 1. A total of 527 articles were located using the given keywords. After the screening was completed, nine articles were found which were duplicates of the others. In assessing eligibility, 453 articles were disqualified because they addressed animal tests, in vitro, in silico, reviews, and theoretical articles. A total of 65 articles were screened again, and it was determined that 52 articles did not match the topic of metabolic syndrome. The number of articles that addressed the topic of discussion was 13 articles, which were used for systematic review (Table 2).

### 3.2. *Spirulina* on Body Weight

Administration of *Spirulina platensis* and *S. maxima* in capsule or tablet form can reduce body weight, BMI, and waist circumference in obese and overweight people and people with high blood pressure. Administration of this microalgae in an amount of up to 266 mg per day has been shown to improve BMI (placebo = −0.86 ± 1.78 vs. *Spirulina* = −0.91 ± 3.62), improved fat mass (placebo = −0.30 ± 4.47 vs. *Spirulina* = −2.19 ± 9.24), and abdominal circumference (placebo = −1.45 ± 2.47 vs. *Spirulina* = −3.21 ± 4.72), although not really yet [13]. Administration of at least 1 g of *Spirulina* per day can reduce body weight in obesity (placebo = −0.3 kg vs. *Spirulina* = −1.6 kg) [19] and BMI in obesity (*p*=0.003) [15], whereas it changes BMI obese (placebo = −0.15 vs. *Spirulina* = −0.5) and dyslipidemia groups (placebo = 0.0 vs. *Spirulina* = −0.4) [19]. Yousefi [31] reported that the administration of this microalga reduced body weight (placebo = −1.45 ± 1.06 kg vs. *Spirulina* = −3.22 ± 1.97 kg; *p* < 0.001) and improved BMI (placebo = −0.63 ± 0.68 kg vs. *Spirulina* = −1.23 ± 0.79 kg; *p* < 0.05) on obesity. *Spirulina* administration significantly reduces weight (*p*=0.007) and BMI (*p*=0.005). This microalga reduces body weight, BMI, and abdominal circumference by reducing visceral fat, macrophage infiltration, fat accumulation in the liver [19, 27, 31, 32], and appetite [18].

### 3.3. *Spirulina* on Hypertension

Administration of *S. maxima* in capsule form reduces blood pressure and hardness index in obese and hypertensive people. Two active ingredients from this microalga play a role in lowering blood pressure, namely, phycocyanin and tripeptide Ile-Gln-Pro [30, 32]. Giving this microalga as much as 266 mg, a day reduces blood pressure (diastolic blood pressure (DBP) (mmHg) = placebo 7.44 ± 12.96 vs. *Spirulina* 3.28 ± 12.28 and systolic blood pressure (SBP) (mmHg) = placebo −4.23 ± 9.39 vs. *Spirulina* −4.20 ± 10.62); however, it is not statistically significant [13], while giving 2 g a day decrease blood pressure (SBP (mmHg) = placebo 151 ± 9.0 vs. *Spirulina* 143 ± 9.0; *p* ≤ 0.001; DBP (mmHg) = placebo 86 ± 7.0 vs. *Spirulina* 79 ± 9; *p* ≤ 0.001), and administration 4.5 g daily decrease SBP (mmHg) (placebo = 140.00 ± 6.05 vs. *Spirulina* = 126.50 ± 5.53; *p* < 0.05) [30], and increase blood vessel flexibility/stiffness index (stiffness index (m/s) = placebo 7.2 ± 0.4 vs. *Spirulina* 6.9 ± 0.7; *p* ≤ 0.001) [32]. The decrease in blood pressure and the increase in stiffness index by both bioactive microalgae are through increased eNOS expression, ACE activity inhibition, and NO production [30, 32].

### 3.4. *Spirulina* on Hyperglycemia

Administration of *S. maxima* and *S. platensis* in capsule form can reduce fasting blood glucose levels and improve insulin sensitivity in people with obesity, overweight, diabetes mellitus, and hypertension. This microalga significantly reduced FBS between placebo and *Spirulina* (*p*=0.02) [27] (placebo 161.27 ± 7.05 vs. *Spirulina* 136.33 ± 4.42; *p* < 0.0001) on Type 2 diabetic people [26]. The bioactive substance in this microalga that improves the blood sugar profile is phycocyanin [18, 26]. The improvement in profile by this microalga is at least 1 g per day and for at least 60 days [20, 26, 27], while despite the administration of relatively large amounts (4.8 g) in one day, no improvement was shown in blood sugar profile due to the short administration of 17 days [28]. The improvement in blood sugar profile due to microalgae administration in some people with metabolic disorders is attributed to phycocyanin. This pigment can increase insulin secretion and sensitivity [20, 26].

### 3.5. *Spirulina* on Hyperlipidemia

Administration of *S. maxima* and *S. platensis* in the form of capsules, liquids, and sauces can reduce dyslipidemia in people with dyslipidemia, diabetes mellitus, and NAFLD [8, 15, 26, 33]. This microalga improves the lipid profile by at least 20 mg daily in liquid form, 2 g in capsule or sauce form, and 6 g in powder form. Bioactive from this microalga that improves blood lipid profile are phycocyanin, gamma linoleic acid, and glycolipid H-b2. The improvement of blood lipid profile by these three bioactive microalgae is through an increase in lipoprotein lipase and pancreatic lipase, a decrease in cholesterol and triglyceride synthesis, and an increase in HDL synthesis [8, 15, 19, 26, 33, 34].

## 4. Discussion

The results of this review were obtained through data analysis from various articles published up to 2023. As the primary debate, 13 articles on the subject of *Spirulina* supplementation were utilized. The results show that *Spirulina* supplementation improves metabolic syndrome in humans. With consumption at a dose of 20 mg to 6 g and within a period of 14 days to 1 year, *Spirulina* supplementation is effective in the treatment of metabolic syndrome. Obesity, hypertension, hyperglycemia, and hyperlipidemia are among them.

*Spirulina* supplementation affects body weight and BMI by reducing fat content and limiting lipid accumulation and appetite [11, 13, 19, 29, 31]. *Spirulina* reduces lipid accumulation in the liver by reducing macrophage infiltration of visceral fat [35]. Phycocyanin from *S. maxima* extract decreased the adipogenic proteins C/EBPα, PPARγ, and aP2, as well as the lipogenic proteins SREBP1, ACC, FAS, LPAATβ, Lipin1, and DGAT1 in 3T3-L1 and C3H10T1/2 cells. These proteins contribute to increased fat accumulation; fat accumulation is low when their expression is suppressed [36]. The role of essential amino acids in spirulina, such as L-phenylalanine, is one of the factors for weight loss by influencing neurotransmitters such as dopamine and norepinephrine [37]. In appetite suppression, phenylalanine concentration increases the release of cholecystokinin [31]. Phenylalanine plays a role in increasing the feeling of satiety by inhibiting gastric emptying. This amino acid can increase cholecystokinin secretion, with the secretion of this hormone inhibiting gastric emptying to reduce energy intake and blood glucose levels [38].

In hypertension, *Spirulina*, which contains the bioactive component, inhibits angiotensin-converting enzyme I and activates angiotensin-converting enzyme II, and also increases the expression of endothelial nitric oxide synthase (eNOS) [20, 30]. The reduction in SBP and DBP is due to the stimulation of phycocyanin in *Spirulina* [13, 32, 33]. This pigment is present in spirulina and causes a decrease in blood pressure levels by promoting the production of eNOS. A study of phycocyanin administration over 25 weeks in spontaneous hypertensive rats (SHR) showed that administration lowered blood pressure by increasing eNOS expression and that the nitric oxide produced could relax blood vessels [39]. A tripeptide from *Spirulina*, namely, Ile-Gln-Pro (IQP), also shows antihypertensive effects. The peptide can inhibit angiotensin-converting enzyme I and activate angiotensin-converting enzyme II. Treatment with the tripeptide in SHR rats at 10 mg/kg per day for 14 weeks [40] and 8 weeks [41] lowers the blood pressure of these rats by inhibiting angiotensin-converting enzyme I and activating angiotensin-converting enzyme II.

In hyperglycemia, treatment with spirulina reduced blood glucose concentrations. Phycocyanin has the potential to act as a protein that mimics insulin or stimulates Langerhans B cells by increasing insulin synthesis. The pigment also improves insulin sensitivity. Administration of phycocyanin at 200 mg/kg over 4 weeks increased insulin secretion in diabetic mice with alloxan induction. The pigment increases the Ca^2+^ and K^+^ channels opening on the pancreatic beta cell membrane, stimulating insulin release [42]. Another study showed that treating KKAy mice with 100 mg/kg for 3 weeks improved insulin sensitivity. The increased glucose uptake occurs by shifting the Glu transporter to the cell membrane by activating the IRS/PI3K/Akt pathway [43].

*Spirulina* is known to reduce the body's lipid profile by increasing lipoprotein lipase and pancreatic lipase, decreasing cholesterol and triglyceride synthesis, and increasing HDL synthesis [8, 17, 19, 26, 33, 34]. The bioactive compounds of this microalgae that play a role in improving the blood lipid profile include phycocyanin, gamma-linolenic acid, and glycolipid H-b2. Administration of phycocyanin 50 mg/kg for 30 days was able to decrease total triglycerides, total cholesterol, and LDL and increase HDL [44], while administration of phycocyanin 1.25% to hamsters fed a hypercholesterolemic diet for 2 months was able to lower TG, T-Chol, and LDL (increased mRNA level of LDL receptor) [45]. Administration of 2% GLA for 8 weeks lowered TG and cholesterol in hypercholesterolemic rats [46]. This essential fatty acid can lower cholesterol 170 times more than linoleic acid [47]. At a dose of 250 mg/kg, this compound can lower the TAG level of hypercholesterolemic mice by inhibiting pancreatic lipase activity [48]. Glycolipid H-b2, a type of glycolipid found specifically in spirulina, also plays a role in lowering hypercholesterolemia in mice.

## 5. Limitations

The review highlights several challenges in determining the optimal dosage and form of *Spirulina* for treating metabolic syndrome. Firstly, the studies used a wide range of dosages (20 mg–6 g) and forms (capsules, tablets, liquids, and sauces), making establishing a consistent treatment protocol difficult. Secondly, the trial durations varied significantly, from as short as 17 days to as long as 1 year, with shorter durations possibly insufficient to observe long-term effects. Additionally, the participant characteristics varied, including healthy individuals and those with metabolic syndrome, diabetes, hypertension, and obesity, affecting the results' generalizability. The review also included both randomized and nonrandomized clinical trials, with nonrandomized trials being more prone to bias, impacting the reliability of the findings. Moreover, there was variability in the outcomes measured, such as BMI, waist circumference, blood pressure, fasting blood glucose, and lipid profiles, complicating direct comparisons or meta-analyses. Inconsistent results were reported, with some studies showing significant benefits of *Spirulina* supplementation. In contrast, others did not observe substantial changes, such as no significant impact on plasma blood glucose in overweight individuals despite high dosages. Lastly, the review concludes that further research is needed to determine *Spirulina*'s optimal dosage and form. By addressing these limitations, future studies can better clarify the role of *Spirulina* in managing metabolic syndrome and provide more definitive recommendations for its use.

## 6. Strengths

The strengths of this study are numerous and noteworthy. Firstly, the study's comprehensive scope ensures that it captures a wide range of data and insights by covering a broad spectrum of studies on *Spirulina* supplementation, considering variations in dosage, trial duration, and participant demographics. This comprehensive approach enhances the robustness and applicability of the findings across different populations and settings. Secondly, the rigorous methodology employed in the review, which involves systematic searches in reputable databases like Scopus and PubMed, enhances the credibility and reliability of the study by ensuring the inclusion of relevant and high-quality studies. Thirdly, the large initial pool of 527 articles narrowed down to 13 suitable human clinical studies indicates a thorough and careful selection process, further ensuring the relevance and rigor of the included studies. Additionally, the diverse range of dosages (20 mg–6 g) and treatment durations (17–360 days) covered in the included studies aids in understanding the effects of *Spirulina* across different usage patterns and provides insights into optimal dosages and treatment periods for various metabolic conditions. By focusing specifically on human clinical studies, the review ensures that its findings directly apply to human health, increasing the practical relevance of the conclusions drawn. Furthermore, the review highlights *Spirulina*'s rich nutritional and bioactive composition, which includes macro- and micronutrients, vitamins, minerals, and bioactive compounds, supporting its multifaceted benefits in improving metabolic syndrome. The findings also indicate multiple health benefits of *Spirulina* supplementation, such as reductions in overweight, obesity, BMI, hypertension, hyperglycemia, and dyslipidemia, making it a valuable supplement for managing metabolic syndrome holistically. The review provides specific mechanistic insights into how components like tripeptides, gamma-linolenic acid, and glycolipids contribute to lowering blood pressure and improving lipid profiles, enhancing the scientific foundation for the observed health benefits. Lastly, the overall findings demonstrate consistent positive clinical outcomes. *Spirulina* supplementation significantly improves various aspects of human metabolic syndrome, thereby strengthening the evidence supporting its efficacy in managing this condition.

## 7. Conclusion

*Spirulina* supplementation in humans has a positive effect on metabolic syndrome. It is due to phycocyanin, Ile-Gln-Pro, gamma-linolenic acid, and the glycolipid H-b2. *Spirulina* supplementation may benefit weight management, blood pressure placebo, blood sugar, and lipid profile regulation in humans. *Spirulina* may help reduce body weight and BMI by decreasing fat accumulation and appetite. It appears to achieve this by influencing fat-storing proteins and hormones involved in satiety. *Spirulina* contains components that inhibit enzymes that raise blood pressure and promote nitric oxide production, a molecule that relaxes blood vessels. This may contribute to lowering blood pressure. *Spirulina* may help regulate blood sugar by stimulating insulin production. It may also improve insulin sensitivity, allowing cells to absorb glucose more effectively. *Spirulina* contains pigment and bioactive that inhibit enzymes that promote bad cholesterol and induce good cholesterol production. However, the form of use, dosage, and treatment should be further researched to determine the right amount for each metabolic syndrome problem.

## Figures and Tables

**Table 1:** Eligibility criteria of included studies based on participants, intervention, comparator, and outcomes (PICO).

Inclusion	Exclusion
*Participants (P)*	*Healthy person*
• Clinical patients with a history of health problems, namely, metabolic syndrome	• Patients with nonmetabolic syndrome
• Metabolic syndrome includes overweight or obesity, hypertension, hyperglycemia, and hyperlipidemia	• Patients taking drugs or health supplements
*Intervention (I)*	• Pregnant and lactating mother
• Various species of *Spirulina* from any region	• Patients with hypersensitivity or allergic to *Spirulina*
• Administration of *Spirulina* doses using various forms and doses	• Study design out of clinical metabolic syndrome topics, such as in vivo, in vitro, in silico, review article, and theoretical article
*Comparator (C)*	
• Placebo or any other medical procedure or mode of therapy	
*Outcome (O)*	
• Make a positive reduction in blood pressure, blood sugar, triglyceride (TG), low-density lipoprotein (LDL), total cholesterol, and body weight	
• Make a positive increase in insulin sensitivity and HDL	
• Potentially reduce chronic effects caused by metabolic syndromes	
*Study design (S)*	
• Clinical trials (randomized or nonrandomized)	

**Table 2:** The result of the *Spirulina* supplementation review on humans.

Author	Year	Location	Design	Duration of trial	Inclusion criteria	Supplementation form species	Dose	Participant	Age (years)	Result
Rajabzadeh et al. [26]	2023	Iran	Randomized, double-blind, placebo-controlled	90 days	Type 2 diabetic mellitus patient	*Spirulina platensis* capsules	2 g/day	*n* = 68 men and women	25 to 50	*Spirulina* supplementation reduced fasting blood sugar, triglycerides, total cholesterol, and LDL-C levels and increased HDL-C levels in Type 2 diabetic patients
Mohammad et al. [27]	2022	Iran	Single-blind, quasiexperimental	60 days	Overweight and obese men, BMI > 25 kg/m^2^	*Spirulina* capsules	1 g/day	*n* = 60 men	30 to 55	*Spirulina* supplementation decreased weight and fasting blood sugar levels in overweight and obese males
Koite et al. [8]	2022	France	Controlled, randomized, double-blind, placebo-controlled	90 days	Patient with metabolic syndrome	*Spirulina platensis* liquid	20 mg/day	*n* = 40 22 men 18 women	18 to 65	Spirulysat did not change the plasma triglycerides, cholesterol, and LDL-C and boosted HDL-C in metabolic syndrome patients
van den Driessche et al. [28]	2020	Netherlands	Randomized, placebo-controlled, double-blind	17 days	BMI 18–30 kg/m^2^	*Spirulina* capsules	4.8 g/day	*n* = 35 15 men 20 women	18 to 70	*Spirulina* consumption did not lower plasma blood glucose in overweight individuals. At the same time, daily consumption in non-hypercholesterolemic men and women also did not affect plasma indicators of intestinal cholesterol absorption or serum lipid concentrations
Gomez-Tellez et al. [13]	2020	Mexico	Double-blind randomized controlled trial	90 days	Abdominal obesity patient	*Spirulina maxima* capsules	266 mg/day	*n* = 50 men and women	18 to 60	*S*. *maxima* supplementation made significant effects on the systolic blood pressure in obese people
Hernández-Lepe et al. [15]	2019	Mexico	Double-blind, randomized, crossover, controlled trial	90 days	Adults with BMI > 25 kg/m^2^	*Spirulina maxima* capsules	4.5 g/day	*n* = 52 men	26 ± 5	*Spirulina maxima* supplementation enhances BMI and HDL, and also decrease total cholesterol, triglycerides, and LDL cholesterol in males with excess body weight and dyslipidemia
Hernández-Lepe et al. [19]	2019	Mexico	Randomized, crossover controlled trial	45 days	BMI ≥ 25 kg/m^2^	*Spirulina maxima* capsules	4.5 g/day	*n* = 52 men	18 to 35	*Spirulina maxima* supplementation improves the BMI and HDL but decrease total cholesterol, triglycerides, and LDL in men with extra body weight and dyslipidemia
Hernández-Lepe et al. [29]	2018	Mexico	Randomized, double-blind, placebo-controlled, and counterbalanced crossover trial	90 days	BMI ≥ 25 kg/m^2^	*Spirulina maxima* capsules	4.5 g/day	*n* = 52 men	26 ± 5	*Spirulina maxima* supplementation improves the body fat percentage in overweight, but mostly in individuals with obesity
Martínez-Sámano et al. [30]	2018	Mexico	Single-blind, randomized, placebo-controlled	90 days	Systemic arterial hypertension patient	*Spirulina maxima* capsules	4.5 g/day	*n* = 16 men and women	20 to 60	On *Spirulina* supplementation, there were significantly reductions in systolic blood pressure but insignificantly in diastole blood pressure
Yousefi, Mottaghi, and Saidpour [31]	2018	Iran	Randomized, double-blind, placebo-controlled clinical trial	90 days	BMI 25–40 kg/m^2^	*Spirulina platensis* tablets	2 g/day	*n* = 38 men and women	20 to 60	*Spirulina* supplementation significantly reduced body weight, waist circumference, body fat, and BMI
Zeinalian et al. [18]	2017	Iran	Randomized double-blind placebo-controlled trial	90 days	Obese individuals, BMI ≥ 30 kg/m^2^	*Spirulina* capsules	1 g/day	*n* = 64	20 to 50	*Spirulina* supplementation had a decrease in BMI and appetite
Szulinska et al. [20]	2017	Poland	Randomized, double-blind placebo-controlled study	90 days	Individuals with hypertension and obesity, BMI ≥ 30 kg/m^2^	*Spirulina maxima* capsules	2 g/day	*n* = 50 25 men 25 women	25 to 60	*Spirulina* supplementation significantly reduced blood glucose and increased insulin sensitivity
Miczke et al. [32]	2016	Poland	Double-blind, placebo-controlled, randomized trial	90 days	Hypertension patient	*Spirulina maxima* capsules	2 g/day	*n* = 40 21 men 19 women	40 to 60	*Spirulina* supplementation had a significantly reduction in SBP and stiffness index

## Data Availability

All data and references used in this study are fully available in the manuscript.
